# Uncovering the Social Deficits in the Autistic Brain. A Source-Based Morphometric Study

**DOI:** 10.3389/fnins.2016.00388

**Published:** 2016-08-31

**Authors:** Alessandro Grecucci, Danilo Rubicondo, Roma Siugzdaite, Luca Surian, Remo Job

**Affiliations:** ^1^Department of Psychology and Cognitive Sciences, University of TrentoTrento, Italy; ^2^Center for Mind/Brain Sciences, University of TrentoTrento, Italy; ^3^Department of Experimental Psychology, Faculty of Psychological and Pedagogical Sciences, Ghent UniversityGhent, Belgium

**Keywords:** autism, morphometric analysis, social deficits, neuroscience, developmental disabilities

## Abstract

Autism is a neurodevelopmental disorder that mainly affects social interaction and communication. Evidence from behavioral and functional MRI studies supports the hypothesis that dysfunctional mechanisms involving social brain structures play a major role in autistic symptomatology. However, the investigation of anatomical abnormalities in the brain of people with autism has led to inconsistent results. We investigated whether specific brain regions, known to display functional abnormalities in autism, may exhibit mutual and peculiar patterns of covariance in their gray-matter concentrations. We analyzed structural MRI images of 32 young men affected by autistic disorder (AD) and 50 healthy controls. Controls were matched for sex, age, handedness. IQ scores were also monitored to avoid confounding. A multivariate Source-Based Morphometry (SBM) was applied for the first time on AD and controls to detect maximally independent networks of gray matter. Group comparison revealed a gray-matter source that showed differences in AD compared to controls. This network includes broad temporal regions involved in social cognition and high-level visual processing, but also motor and executive areas of the frontal lobe. Notably, we found that gray matter differences, as reflected by SBM, significantly correlated with social and behavioral deficits displayed by AD individuals and encoded via the Autism Diagnostic Observation Schedule scores. These findings provide support for current hypotheses about the neural basis of atypical social and mental states information processing in autism.

## Introduction

Autism Spectrum Disorder (ASD) is a category of pervasive developmental disorders (PDD) that affect 1 in 150 children (Rapin and Tuchman, 2008). Autistic symptomatology is characterized by severe impairments that mainly affect social interaction, communication, while sparing basic cognitive skills (Misra, 2014), and not implying emotional disturbance (Rapin and Tuchman, 2008). The term ASD was introduced by Allen (1988) and included: autistic disorder (AD), Asperger syndrome (AS), and PDD not otherwise specified (Levy et al., 2009; Pina-Camacho et al., 2012). Recently, the DSM V revised the conceptualization of those disorders and currently diagnostic classifications only include Autism Spectrum Disorders (ASD) (Lord and Bishop, 2015). Nevertheless, the diagnostic criteria adopted in the current study still refer to DSM IV, and we focused primarily on autism disorder (AD). Consequently, we use the words “autism” and “autistic” to specifically refer to AD.

The mind-blindness theory proposed that characteristic problem in social interaction arises because AD have difficulties in mentalizing and understanding psychological dynamics in other people and in oneself (Baron-Cohen et al., 1985; Frith et al., 1991). Support for this model comes from behavioral studies showing that autistics poorly perform on tasks that require theory of mind (ToM) abilities (Baron-Cohen et al., 1985; Misra, 2014). The poor performance of people with autism on ToM task is thought to be due to a conceptual deficit or to processing peculiarities. In normally developing children ToM tasks elicit intuitive social insights into people. In contrast, autistic children treat these tasks as logical-reasoning problems relying on language and non-social cognitive functions (Tager-Flusberg et al., 2009). In line with this hypothesis, functional MRI investigations of AD have highlighted reduced responses in cortical areas related to social interaction (Frith, 2003; Di Martino et al., 2009; Lombardo et al., 2011; Gliga et al., 2014), and the engagement of areas associated with general problem solving abilities (Frith, 2003). Moreover, functional connectivity in a group with autism shows abnormal pattern in areas mediating ToM and in the mirror neuron system (MNS) as well (Fishman et al., 2014; Cheng et al., 2015; Kana et al., 2015).

On the other hand, studies of volumetric changes in the autistic brain resulted in inconsistent brain structures across studies: prefrontal cortex (Courchesne et al., 2011b), cerebellum (Sparks et al., 2002), temporal lobe (Palmen et al., 2006), both the frontal (Herbert et al., 2004; Jiao et al., 2010) and parietal lobes (Courchesne et al., 1993) and amygdala (Schumann et al., 2004). Some inconsistencies in the structural MRI literature may be attributed to differences in methodology, age, heterogeneity of the disorder (Geschwind, 2009), or diffuse structural abnormalities. Diffuse structural abnormalities in autism could reflect impairment of many, if not most, brain networks (Müller, 2007).

Recently, predictive models of autism based on pattern recognition in structural MR images have been successfully developed. Ecker et al. (2010) adopted a support vector machine (SVM) method to discriminate ASD individuals from controls. Brain areas in the temporal lobe, precuneus, hippocampal, and fusiform gyri were crucial for discrimination. Neural abnormalities discovered with such methodologies, however, rarely correlate with clinical criteria, or correlate only with generic total scores, and not with specific subscales (Eliez and Reiss, 2000; Lord et al., 2000; Hardan et al., 2006a,b; Ecker et al., 2010; Griebling et al., 2010). In other words, these correlations may refer more to a general severity of pathology than to specific deficits.

We suggest that neuroanatomical markers of autism disorder may be better understood by studying large-scale anatomical networks (Minshew and Williams, 2007; Schaer et al., 2013). SBM is a data-driven multivariate alternative to the standard Voxel-Based Morphometry (VBM), and it may be particularly suitable to the investigation of anatomical changes in autism. SBM takes into account information across different voxels and identifies unpredicted, naturally occurring patterns of covariance across brain regions (Xu et al., 2009). Notably, such anatomical covariance has been shown to reflect functional connectivity (Evans, 2013). For these reasons, we expect SBM to individuate large anatomical networks of gray-matter which show aberrant patterns of covariance in AD, as compared to control. We also expect those networks to include brain areas that previous studies showed to be anatomically or functionally abnormal.

Given the heterogeneity of AD and the need for large-scale samples of MR and clinical measures, the present study utilizes the ABIDE (Autism Brain Imaging Data Exchange) database.

## Methods

### Participants

Structural MRI of 82 participants (32 AD and 50 controls) were extracted from Autism Brain Imaging Data Exchange (ABIDE). Details of acquisition, informed consent, site-specific protocols, specific diagnostic criteria for each data set can be found at http://fcon_1000.projects.nitrc.org/indi/abide/index.html. The following committees approved the protocols of each site: the Human Subjects Protection Committee of the California Institute of Technology (CAL), the Institutional Review Board at the University of Pittsburgh (PBG) and the University of Utah School of Medicine (USM).

From around 3000 subjects available, we carefully selected participants by gender (males), age (range: 18–39 years old), as well as parameters of the MR scanners (image type: T1; magnetic field strength: 3T). This first selection resulted in a dataset composed by structural MRI of 283 subject acquired with 12 different MRI scanners. Next, the 283 subjects were screened on the basis of the DSV-IV for either the absence of any neuropsychiatric disorders (control group), or the diagnosis of Autism (AD) (patients group). Further inclusion criteria were: (a) the indication of the IQ score computed on the basis of Wechsler abbreviated scale of intelligence (WASI); (b) scores on at least three specific subscales of the Autism diagnostic observation schedule (ADOS) for AD participants. Finally, individuals with any other pathology or comorbidity were rejected. An in house made Dori script iteratively sampled the database until controls and AD group were balanced for age and there was no association between groups and a particular scanner.

This procedure yielded a dataset comprising 82 participants (32 AD and 50 controls) tested on one of the two MR scanners, with the same MR sequence (Table 1 in Supplementary Material). The scanners belonged to the same vendor (i.e., Siemens MAGNETOM TrioTim, Siemens MAGNETOM Allegra), and participants were equally distributed between scanners (See Table 1 in Supplementary Material). The two groups did not differed for age [*t*_(31)_ = 0.2953, *p* = 0.7728]. However, there was a difference in IQ scores [*t*_(31)_ = 2.9586, *p* = 0.0041]. See Table 1 in Supplementary Material. This is in line with previous observations for which the prevalence of mental retardation in autism is ~60% groups, and AD naturally differ from normal controls for IQ (Amaral et al., 2008).

### Data analysis

Source-based morphometry (SBM) is a multivariate technique that takes advantage of independent component analysis (ICA) (Lee, 1998; Xu et al., 2009). ICA is a statistical technique that is widely used in many fields of biomedical research for signal analysis. In the field of neuroscience ICA has found important application in EEG/MEG/fMRI data analyses to isolate noisy artifacts.

Pulling images from different MR scanners is known to lead to confounding, even though MR sequences are the same (Han et al., 2006). This is especially true for a massive univariate analysis, such as VBM. However, SBM can decompose the MR signal in several maximally independent sources, or independent components (ICs). In this study, few sources may reflect signal differences among MR scanners, while the majority of ICs individuate networks of gray-matter that share patterns of covariance among subjects (Xu et al., 2009; Kaspárek et al., 2010; Kubera et al., 2014). Artifactual components are usually easy to detect because are asymmetric, do not follow the anatomical organization of the brain, and do not exhibit any coherent patterns. In addition, the distribution of subjects throughout scanners is similar in both controls and AD, and this should guarantee that group differences are not due to artifact components.

The preprocessing of images is identical to the procedure adopted for classical VBM analyses. Brain extraction and robust center estimation was automatically carried out using FSL Brain extraction tool (BET) (Smith, 2002). For normalization and segmentation we used the SPM toolbox VBM8. Images were spatially normalized to the 152 average T1 MNI (Montreal Neurological Institute) template, and segmented into gray-matter (GM), white-matter (WM), and cerebrospinal fluid (CSF). The normalized gray-matter images were smoothed with 8-mm full width at half-maximum (FWHM) Gaussian kernel to establish spatial correspondence between the different brains.

Source-based morphometry analysis was carried out using the GIFT toolbox (http://icatb.sourceforge.net) (Xu et al., 2009). The minimum description length (MDL) principle was used to estimate a number of independent components. MDL found eight reliable ICs. We performed ICA using a neural network algorithm (Infomax) that attempts to minimize the mutual information of the network outputs to identify naturally grouping and maximally independent sources (Bell and Sejnowski, 1995). ICA was repeated 20 times in ICASSO (http://research.ics.aalto.fi/ica/icasso/) and the resulting components were clustered to ensure the consistency and reliability of the results. Reliability is quantified using a quality index Iq, ranging from 0 to 1 and reflecting the difference between intra-cluster and extra-cluster similarity (Himberg et al., 2004). All the 8 components extracted from the GM images were found to be associated with an Iq > 0.97 indicating a highly stable ICA decomposition.

SBM involves converting each gray-matter volume into a vector. As a result, we obtained a matrix where the 82 rows represent the 82 subjects (the first 50 rows represent controls, while the other 32 AD), and each column indicates a voxel. This matrix was decomposed into two matrices by ICA. The first matrix is named “mixing matrix” and it is composed by a subject per row and an IC per column. Therefore, the mixing matrix indicates how much a subject expresses a given component. For this reason, values in the mixing matrix are called “loading coefficients.” The second matrix is named source matrix and it specifies the relation between the ICs and the voxels. For gray-matter volume component visualization the source matrix was reshaped back to a three-dimensional image, scaled to unit standard deviations (Z maps) and thresholded at *Z* > 2.5.

We used the mixing matrix to verify whether components are differently expressed between controls and AD. A two sample *t*-test without assuming equal variances (*F*-test revealed unequal variances) was used to test whether all the ICs are similarity expressed by either of the groups. Similarly, we used the loading coefficients in the mixing matrix to test a linear relation among ADOS scores and the level of components' expression. All the results were thresholded at *p* < 0.05 corrected for Family Wise Error (FWE).

## Results

**Figure 1:**
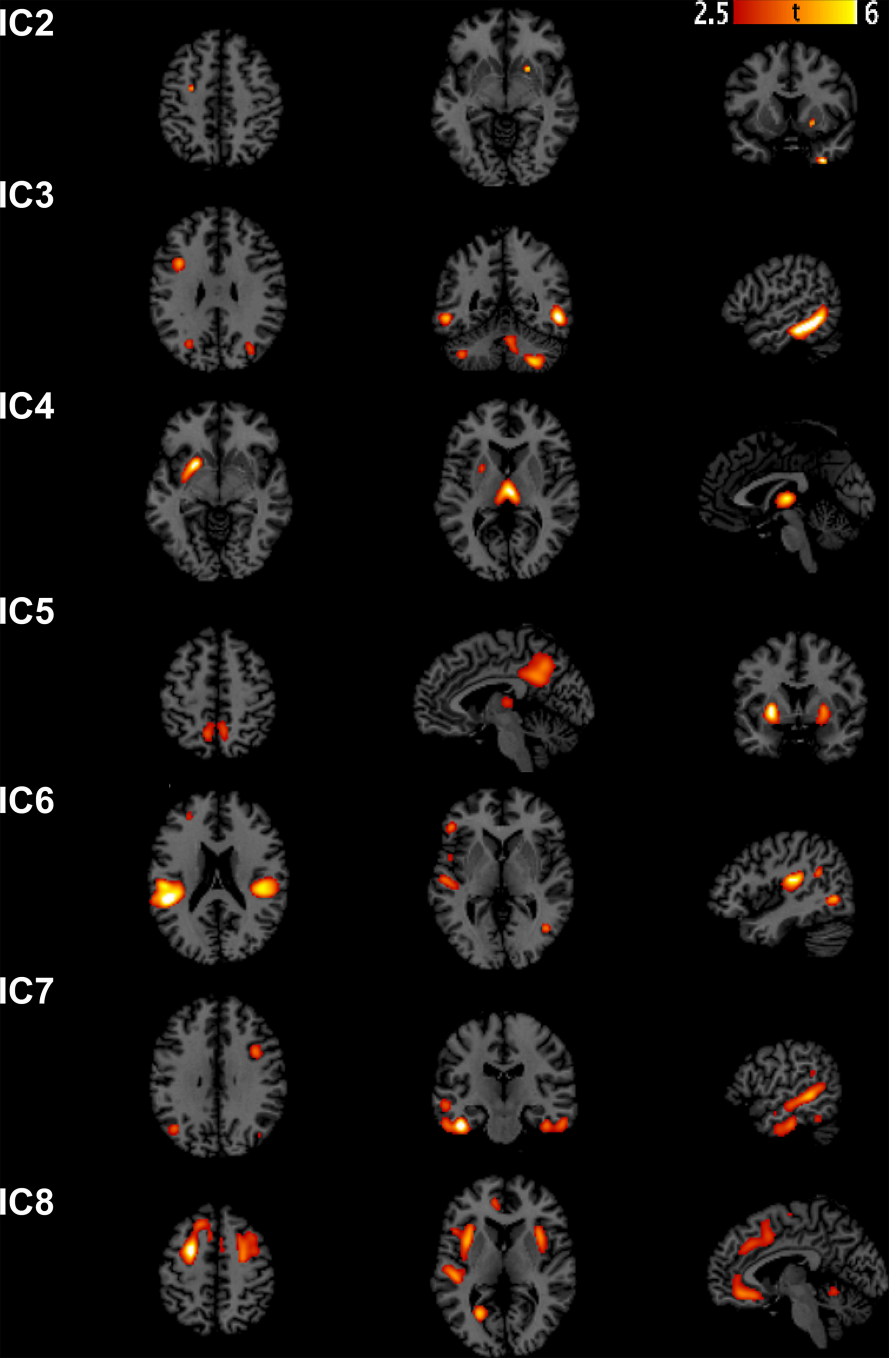
**Sources discovered by SBM**. According to the estimation of the number of components, eight independent components were extracted. Note that IC1 is not graphically represented because no voxel survived after thresholding for *Z* > 2.5.

**Figure 2:**
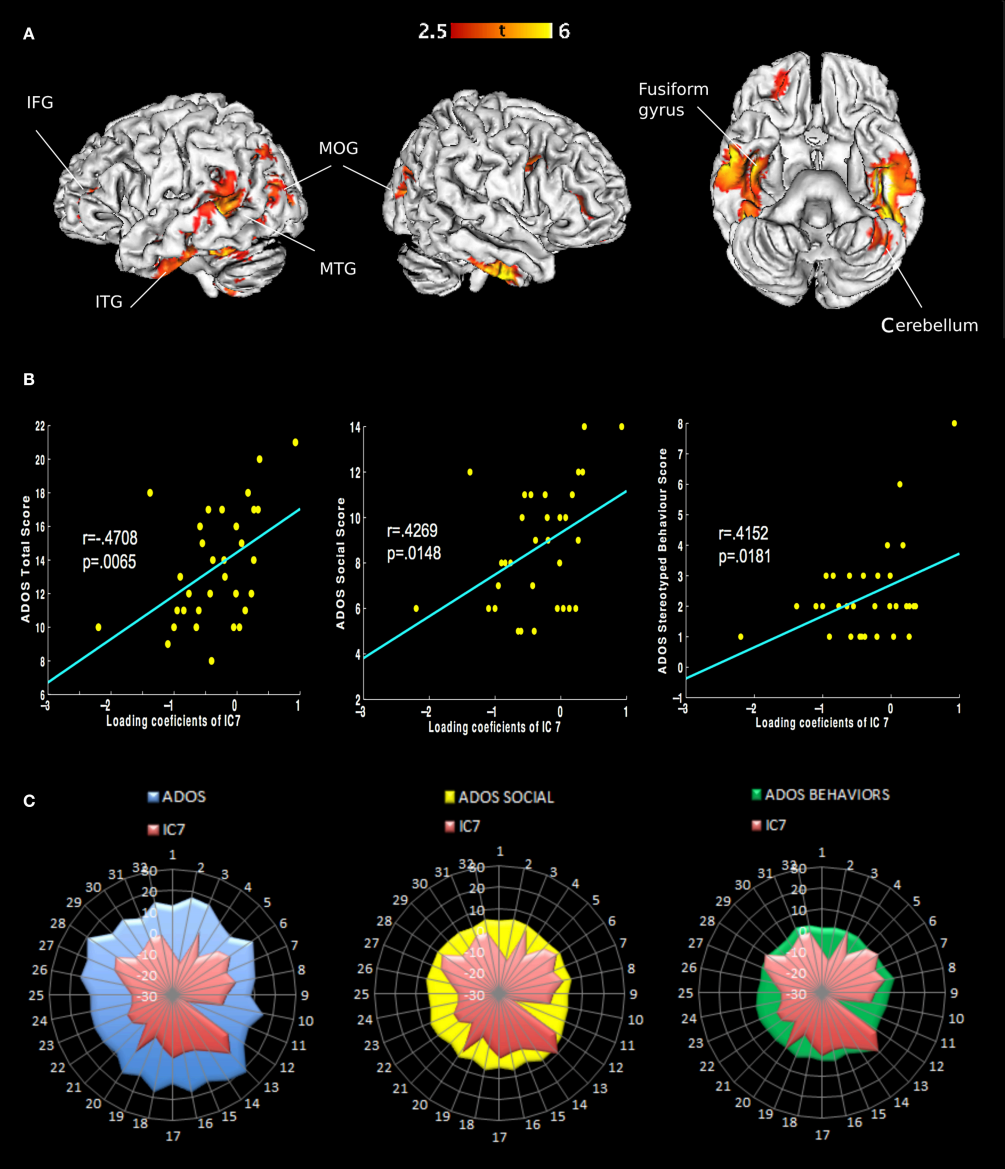
**Representation of ASN in the brain and behavior in AD. (A)** Only the seventh component shows different loading coefficient between ASD and controls. The regions involved are: inferior frontal gyrus, middle frontal gyrus, superior frontal gyrus, inferior temporal gyrus, middle temporal gyrus and superior temporal gyrus, fusiform gyrus, parahippocampal gyrus, paracentral lobule, precuneus and cerebellar tonsil (Table 2 in Supplementary Material). **(B)** Correlations of behavioral measures with loading coeficients values in IC 7: Classic Total ADOS Score (communication subscore + social interaction subscore); Social Total subscore: Stereotyped behaviors and restricted interest. **(C)** Covariance plots showing each subjects' ADOS scores and individual ASN loading coefficients.

We extracted eight independent components (Figure 1). However, only the 7th component was significantly different [*t*_(31)_ = 2.9482, *p*_(FWE)_ = 0.0042] between AD and controls. We call this component “autism-specific structural network” (ASN). Anatomical labels of the regions composing ASN were obtained using the WFU PickAtlas (Tzourio-Mazoyer et al., 2002). Among regions that differ between AD and controls we found: inferior, middle, superior temporal gyri, fusiform gyrus, parahippocampal gyrus, paracentral lobule, precuneus, cerebellar tonsil, and portions of the inferior, middle, and superior frontal gyri (Figure 2). All the gray-matter regions of ASN are presented in Table 2 in Supplementary Material, and in Figure 2.

To ensure that differences in IQ (see Table 1 in Supplementary Material) did not account for brain differences we correlated ASN loading coefficients (i.e., 7th column of the mixing matrix) against IQ values. The correlation was not significant (*p* = 0.6336), thus excluding the relevance of IQ on this component.

Notably, ASN significantly correlated with the total scores of ADOS, and also with two ADOS subscales measuring highly relevant impairments in AD, i.e., difficulties in social interactions and stereotyped behavior. Both variables show significant correlation with the loading coefficient of ASN, after Bonferroni correction for multiple comparisons. Classic Total ADOS Score: *r* = 0.4708, *p*_(FWE)_ = 0.0065. Social Total subscore: *r* = 0.4269, *p*_(FWE)_ = 0.0148. Stereotyped behaviors and restricted interest: *r* = 0.4152, *p*_(FWE)_ = 0.0181. See Figure 2.

## Discussion

In this study we presented for the first time a whole brain morphometric method (SBM), based on independent component analysis, which shows alterations in gray-matter between AD individuals and controls. This innovative multivariate procedure was applied to detect brain networks that exhibit abnormal pattern of gray-matter covariance in AD. We showed that morphometric changes in autism, as detected by SBM, are significantly associated with observable social and behavioral deficits (ADOS scores).

SBM addresses a different question in comparison with massive univariate approaches to morphological changes, as Voxel-Base-Morphometry (VBM) or ROI differences between groups. Univariate approaches detect voxel- or ROI-based changes in gray-matter concentrations, while SBM individuates different levels of expression in maximally independent gray-matter networks. Differences in network expression in turn implicate differences in gray-matter concentrations, distributed along patterns of voxel-covariation. SBM is particularly suitable to study autism as spectrum disorder, since anatomical changes are likely to be distributed along networks of brain regions. In this scenario, such anatomical changes may not be locally detectable by univariate approaches.

In the present study we found an autism-specific structural network (ASN) which covers brain regions found to exhibit functional and structural abnormalities in previous studies compared with controls (Castelli et al., 2002; Saxe and Wexler, 2005; Amaral et al., 2008; Courchesne et al., 2011b; Ecker et al., 2012; Nickl-Jockschat et al., 2012; Pappaianni et al., 2016).

### Autism-specific structural network (ASN)

#### Temporal lobe

The core of ASN is localized in temporal regions involved in processing and integrating social stimuli, such as faces and intention-related movements.

ASN is centered in vast portions of the temporal lobes, suggesting that the temporal lobe may have particular relevance in the autistic disorder. ASN includes parts of the inferior, medial and superior temporal sulci, the fusiform gyrus, and the parahippocampal gyrus. Temporal lobe regions are implicated in social perception, auditory processing, language, and theory of mind. These abilities were shown to be the most damaged in autism (Gendry Meresse et al., 2005). In the present study inferior temporal and fusiform gyri are the brain regions which disclosed the greatest differences between AD and controls. These are the areas involved in high-level visual processing and object recognition, in particular face recognition (Rossion et al., 2003). Furthermore, the posterior superior temporal sulcus (STS) is a core region for perception of social acts (Zilbovicius et al., 2006). STS appears to respond also to biological motion and to how another person's motion is related to his/her intentions (Vander Wyk et al., 2009). Pelphrey et al. (2007) individuated a functional deficit in ASD regarding neural mechanisms for processing emotional facial expressions and biological motion. This system of regions referring to the deficit includes the amygdala, posterior STS and fusiform gyrus. This evidence provides further support for the idea that autism disorder relates to an impairment in processing social-relevant information (Baron-Cohen et al., 1985).

#### Frontal lobe

ASN includes bilateral clusters in both the superior frontal gyrus (BA6) and in the precentral gyrus (BA4). Those areas correspond to premotor, supplementary, and primary motor cortices. Action processing impairments and repetitive movements are commonly observed among subjects affected by autism. Even though motor impairments are not considered one of the main symptoms of ASD, recently increasing attention has been directed to motor aspects aiming to improve a diagnostic process (Torres et al., 2013).

Our results also showed abnormalities in the inferior part of the frontal gyrus, a brain region implied in executive processes and language (Gotts et al., 2012; Libero et al., 2014). Since AD are largely characterized by deficits in imitation, language, ToM and empathy, a theory known as “broken mirror” hypothesis of ASD, has suggested that a dysfunctional MNS is an important factor in AD pathogenesis (Oberman et al., 2005). Sustaining this hypothesis, some recent studies have found abnormal pattern of functional connectivity in networks believed to underlie social abilities, as MNS and ToM systems (Fishman et al., 2014). However, the role of the mirror system in understanding AD symptomatology is still controversial (Grecucci et al., 2013; Hamilton, 2013; Enticott et al., 2014).

### Other areas implicated in autism

Cerebellar pathology is usually reported in autism (Courchesne et al., 1994, 2011a; Cauda et al., 2011; Rogers et al., 2013), both at structural and functional level (Fatemi et al., 2012). We also found cerebellum to be a part of the autism-specific structural network. Evidence suggests that the cerebellum supports cognitive functions, including language and executive functions (Becker and Stoodley, 2013), which are typically damaged in autism.

Previous meta-analyses of VBM in ASD have proposed that the amygdala and the insula are two brain regions frequently associated with abnormalities in autism (Cauda et al., 2011). In our study, we did not find structural changes in those regions. Our interpretation is that the amygdala and the insula are likely to play a role in autism, though these areas belong to a broader limbic system that, considered as a whole, may be sufficiently preserved in AD. Therefore, the neurostructural configuration of the limbic system was not markedly different between AD and controls.

### Correlations with behavioral deficits

Finally, we found that the autism-specific structural network (ASN loading coefficients) significantly correlates with ADOS social and ADOS stereotypic behavior scores. Such evidence further supports the relation between the structural differences in ASN and the behavioral deficits displayed by AD individuals. These findings provide support for current hypotheses about the neural basis of atypical social and mental states information processing in autism.

## Conclusion

Autism is a behaviorally diagnosed pathology, although evidence of brain abnormalities in AD, such as atypical neural “connectivity” (Ecker et al., 2012), are increasing. MRI investigation is a precious tool for shedding light on both the neurological causes and the neurodevelopment of AD. This knowledge is needed to get fast and objective diagnosis, but also for developing appropriate treatments. The present study, along with increasing evidence (Ecker et al., 2010; Jiao et al., 2010), suggests that structural MRI may become a diagnostic instrument useful to improve the traditional behavior-based diagnosis.

## Author contributions

AG: Experimental design, data collection and writing. DR: Experimental design, data collection, analyses and writing. RS: Experimental design, data collection, analyses, and writing. LS: writing of the paper. RJ: writing of the paper.

### Conflict of interest statement

The authors declare that the research was conducted in the absence of any commercial or financial relationships that could be construed as a potential conflict of interest.
