# Supplementary material for: Uncovering the Social Deficits in the Autistic Brain. A Source-Based Morphometric Study
Source: Front Neurosci. 2016 Aug 31;10:388. doi: 10.3389/fnins.2016.00388 (PMC5005369; doi:10.3389/fnins.2016.00388)
Supplement: Supplementary file 1 [file Table1.DOCX]

**Table 1.** **Behavioural characteristics of participants (mean, standard deviation, t-test).**

|  | **AD**  **(n=32)** | **CONTROLS**  **(n=50)** | **t-test**  **AD vs CONTROLS** |
| --- | --- | --- | --- |
| **AGE** | 24.8 (±5.4) | 25.2 (±5.9) | p=0.7728 |
| **IQ** | 106.7 (±13.8) | 115.2 (±12.1) | p<0.05 |
| **GENDER** | all males | all males |  |
| **DSM IV** | Autism disorder (AD) | No history of  psychiatric disorders |  |
| **ADOS Total**  **ADOS Social**  **ADOS Stereotyped behavior** | 13.5 (±3.6)  8.7 (±2.6)  2.3 (±1.5) | N/A  N/A  N/A |  |
| **Data1 CAL**  **Data2 USM**  **Data3 PBG** | 26  12  12 | 16  8  8 | cf. p. 6 |
