# Supplementary material for: Uncovering the Social Deficits in the Autistic Brain. A Source-Based Morphometric Study
Source: Front Neurosci. 2016 Aug 31;10:388. doi: 10.3389/fnins.2016.00388 (PMC5005369; doi:10.3389/fnins.2016.00388)
Supplement: Supplementary file 2 [file Table2.DOCX]

**Table 2. Talairach labels, volume and peak coordinates of the source ASN area.**

| **Area** | **Brodmann**  **Area** | **volume (cc) L/R** | **Random effects:**  **Max Value Talairach (x, y, z) L/R** |
| --- | --- | --- | --- |
| **Fusiform Gyrus** | 20, 36, 37 | 2.8/1.2 | 7.5 (-40, -20, -23)/5.0 (46, -43, -14) |
| **Sub-Gyral** | 20 | 1.0/0.3 | 6.9 (-42, -24, -21)/4.7 (45, -50, -10) |
| **Uncus** | 20 | 0.1/0.1 | 5.9 (-39, -15, -28)/4.7 (37, -10, -34) |
| **Inferior Temporal Gyrus** | 20, 37 | 0.6/1.6 | 5.6 (-45, -42, -17)/5.7 (52, -7, -29) |
| **Parahippocampal Gyrus** | 36 | 0.3/0.0 | 5.3 (-37, -23, -23)/ - |
| **Middle Temporal Gyrus** | 21, 22 | 1.4/0.2 | 5.3 (-52, -38, 2)/3.9 (46, -2, -30) |
| **Superior Temporal Gyrus** | 22 | 0.3/0.0 | 4.4 (-56, -43, 6)/ - |
| **Middle Occipital Gyrus** | 19 | 0.3/0.1 | 4.2 (-40, -73, 16)/4.0 (42, -72, 16) |
| **Culmen** |  | 0.1/0.0 | 4.2 (-45, -45, -19)/ - |
| **Inferior Frontal Gyrus** |  | 0.0/0.1 | - /4.1 (37, 10, 29) |
| **Precuneus** | 39 | 0.1/0.0 | 4.0 (-40, -64, 34)/ - |
| **Cerebellar Tonsil** |  | 0.1/0.0 | 3.5 (-36, -50, -38)/ - |
| **Middle Frontal Gyrus** |  | 0.0/0.1 | - /3.5 (37, 13, 26) |
